# The interaction between kynurenine pathway, suicidal ideation and augmentation therapy with minocycline in patients with treatment-resistant depression

**DOI:** 10.1177/02698811231173588

**Published:** 2023-05-15

**Authors:** Maria Antonietta Nettis, Giulia Lombardo, Caitlin Hastings, Zuzanna Zajkowska, Nicole Mariani, Naghmeh Nikkheslat, Luca Sforzini, Courtney Worrell, Amina Begum, Mollie Brown, Anthony J Cleare, Allan H Young, Carmine M Pariante, Valeria Mondelli

**Affiliations:** 1 Department of Psychological Medicine, Institute of Psychiatry, Psychology and Neuroscience, King’s College London, London, King’s College London, London, UK; 2 National Institute for Health and Care Research Maudsley Biomedical Research Centre, South London and Maudsley NHS Foundation Trust and King’s College London, London, UK; 3 Wellcome Trust, Mental Health Team, Research Programmes, London, UK

**Keywords:** Depression, kynurenine pathway, suicidal ideation, inflammation, minocycline

## Abstract

**Background and aims:**

We investigated kynurenine pathway (KP) metabolites levels and their association with suicidal ideation in patients with treatment-resistant depression (TRD) and elevated peripheral inflammation. The effect of antidepressant augmentation with minocycline on KP metabolites was tested.

**Methods:**

We analysed data from MINocycline in DEPression, a 4-week, randomized, placebo controlled (1:1) trial of minocycline added to antidepressant treatment in 39 TRD patients (*n* = 18 minocycline; *n* = 21 placebo) with C-reactive protein (CRP) ⩾1 mg/L. At baseline and at week 4, we collected data on suicidality (Beck Depression Inventory) and blood samples to measure inflammatory markers and KP metabolites. We tested (1) the association of KP metabolites ratios with inflammatory markers and suicidal ideation at baseline and (2) the role of suicidality and treatment (minocycline vs placebo) in affecting KP changes over time.

**Results:**

At baseline, kynurenine/tryptophan (KYN/TRP) ratio positively correlated with high-sensitivity CRP (Spearman’s ρ = 0.35, *p* = 0.02) and IL-10, (ρ = 0.41, *p* = 0.009); and tumour necrosis factor was positively correlated with quinolinic acid/3-hydroxykynurenine ratio (ρ = 0.55, *p* < 0.001). Moreover, participants with suicidal ideation showed higher levels of KYN/TRP (*U* = 143.000, *p* = 0.02) than those without suicidal ideation. There was no significant effect of minocycline on KP metabolites changes from baseline to week 4. However, in the minocycline group, the number of participants with suicidal thoughts decreased from 44.4% (8/18) to 22.2% (4/18).

**Conclusion:**

Increased KP neurotoxic metabolites are associated with elevated peripheral inflammation in depressed individuals, particularly in those with suicidal ideation. Targeting KP in this population could be a potential effective personalized approach. Whether this includes minocycline should be investigated in future larger trials.

## Introduction

Suicidality is a frequent clinical feature of major depressive disorder (MDD) and a major global health problem. Alongside psychosocial factors, various biological mechanisms have been implicated in increasing the risk of suicide and suicidal behaviour (Pandey, 2013). For example, a dysregulation of the enzymes in the kynurenine pathway (KP) has been found in suicidal patients and suggested as potential mechanism underpinning suicidality (Bryleva and Brundin, 2017a).

The KP is an essential pathway in the metabolism of the serotonin precursor, tryptophan, and is strictly regulated by immune activation. Under normal conditions, a proportion of tryptophan is metabolized to kynurenine, which, in turn, is transformed by astrocytic kynurenine aminotransferases into kynurenic acid (KynA). KynA has been suggested to have neuroprotective effects, including anti-inflammatory mechanisms, reduction in glutamate excitotoxicity and enhancement of synaptic plasticity (Kopra et al., 2021). By contrast, an increased immune activation (i.e. inflammation) leads to the activation of indoleamine 2,3-dioxygenase (IDO-1 and 2), a key enzyme in the metabolism of tryptophan. This results in a reduction in serotonin levels and shifts the activation of KP towards the production of kynurenine neurotoxic metabolites (Nettis, 2021; Sforzini et al., 2019).

KP neurotoxic metabolites include 3-hydroxykynurenine (3HK) and quinolinic acid (QUIN). Prior studies have found that 3HK activates neuronal apoptosis and increases the concentration of oxygen reactive species (Kopra et al., 2021). Similarly, QUIN is capable of forming complexes with iron which leads to the formation of reactive oxygen species (Goda et al., 1996). As reviewed by Bryleva and Brundin (2017b), QUIN also reduces synaptogenesis and protein synthesis, more specifically of brain-derived neurotrophic factor protein. Moreover, it is an agonist of the *N*-methyl-d-aspartate receptor, causing an increase in glutamate release by neurons and inhibition of its uptake and degradation by astrocytes, resulting in overall neurotoxicity (Bryleva and Brundin, 2017b).

As an indication of IDO activation and KP dysregulation, previous works have used the increased kynurenine/tryptophan (KYN/TRP) ratio in plasma. Interestingly, an increased KYN/TRP (with increased KYN levels and lower TRP levels) has been found in individuals with MDD and a history of suicidality when compared with those without such history or with healthy controls (Bradley et al., 2015; Messaoud et al., 2019; Sublette et al., 2011). Moreover, raised QUIN levels and a simultaneous decrease in neuroprotective metabolites have been observed in suicidal patients, in both in vivo and post-mortem studies (Erhardt et al., 2013; Steiner et al., 2011). This results in an imbalance of metabolites that modulate glutamate neurotransmission and neuroinflammation (Bryleva and Brundin, 2017b) and might lead to the development of suicidal ideation and behaviour (Bryleva and Brundin, 2017a).

Overall, the activation of the KP is believed to be an important mechanism linking peripheral inflammation to the onset of depression, through the production of neurotoxic metabolites, neuroinflammation and oxidative stress (Nettis et al., 2021). Therefore, treatment interventions targeting peripheral inflammation and KP could be tested in people with immune-related depression, with a particular focus on suicidality.

Previous preclinical research suggested that anti-inflammatory medications (such as non-steroidal anti-inflammatory drugs), as well as antidepressants with anti-inflammatory effects (e.g. venlafaxine), may exert an antidepressant effect by decreasing the levels of peripheral inflammation and by inhibiting tryptophan 2,3 dioxygenase (the other enzyme which activates the KP) (Dawood et al., 2022). Conversely, the expression of IDO can be reduced by anti-inflammatory drugs that reduce interferon gamma (IFN-γ) production (Pfefferkorn et al., 1986; Yadav et al., 2007) and by nonsteroidal anti-inflammatory drugs that inhibit cyclooxygenase 2 (Prendergast et al., 2014). Even when mechanisms for reducing inflammation occur primarily in the periphery, research has shown that flux through the KP is decreased in the brain (Dawood et al., 2022).

The antibiotic minocycline has been shown to inhibit the KP in preclinical studies. For instance, in rats injected with lipopolysaccharide, the administration of minocycline appeared to block IDO indirectly, by attenuating lipopolysaccharide-induced pro-inflammatory response and thus preventing the development of depressive-like behaviours (O’Connor et al., 2009). Moreover, in a rat model of neuropathic pain, the administration of intraperitoneal minocycline diminished the mRNA levels of IDO2 and kynurenine 3-monooxygenase, the enzyme leading to 3HK production (Rojewska et al., 2018).

Interestingly, minocycline showed the ability to pass the blood–brain barrier, which would facilitate neuroprotective properties (Soczynska et al., 2012). Recent evidence further showed that minocycline modulates pathophysiological processes including oxidative stress, neurogenesis and apoptosis, mitochondrial dysfunction, and neuroinflammation (Husain et al., 2017). Our own group (Nettis et al., 2021) found preliminary evidence of effectiveness of add-on treatment with minocycline in patients with treatment-resistant depression (TRD) and increased levels of peripheral inflammation. However, no study so far has investigated minocycline effect on the KP and suicidal ideation in patients with depression.

With the present work, we aimed to (1) replicate previous evidence on the association between KP and suicidality in a sample of outpatients with TRD selected for increased peripheral inflammation (MINocycline in DEPression (MINDEP) study). We hypothesized that individuals with suicidal ideation would show increased IDO activity, as reflected by increased KYN/TRP ratio and increased levels of neurotoxic metabolites such as QUIN; (2) explore the association between metabolites ratios of the KP, indicating a KP diversion towards neurotoxicity and markers of peripheral inflammation and (3) explore the effect of 4-week treatment with minocycline on suicidality and KP metabolites in the same sample of patients. We hypothesized that minocycline would reduce the number of patients with suicidal ideation from baseline to week 4 of treatment and that this would be mediated by changes in the KP metabolites.

## Methods

MINDEP was a single-centre, randomized (1:1 minocycline/placebo) placebo-controlled, double-blind parallel group trial of adjunctive oral minocycline (200 mg/day) added to ongoing treatment in patients who had failed to respond adequately to at least one antidepressant in the current depressive episode and had elevated peripheral inflammation as shown by C-reactive protein (CRP) levels ⩾1 mg/L. This is a CRP threshold that, according to a recent meta-analysis, defines ‘elevated levels of CRP’ that are present in around 60% of depressed patients (Osimo et al., 2019).

Of note, the minocycline dose of 200 mg/day was the most consistently used (and proved to be safe) in prior trials in patients with MDD (Cai et al., 2020; Rosenblat and McIntyre, 2018).

All visits took place at the Clinical Research Facility of King’s College Hospital, London. Data from the same randomized clinical trial (RCT) on the longitudinal antidepressant effect of minocycline according to baseline levels of inflammation have been previously published (Nettis et al., 2021).

### Inclusion and exclusion criteria

Inclusion and exclusion criteria for the participants in the trial are described below.

*Inclusion criteria*: (1) aged 25–60, with a current Diagnostic and Statistical Manual of Mental Disorders (DSM-5) diagnosis of non-psychotic MDD; (2) non-responders to the current antidepressant taken at therapeutic doses, for at least 6 weeks, as indicated by a current score of at least 14 on the 17-item Hamilton Depression Rating Scale (HAM-D); (3) tolerant to the current antidepressant and accepting augmentation with minocycline; (4) having the ability to understand and sign a written informed consent form prior to participation in any screening procedures; (5) having CRP levels ⩾1 mg/L at the screening visit and (6) no changes in current therapy planned to occur in the weeks following the enrolment in the study.
*Exclusion criteria*: (1) active suicidal ideation of significant concern to require intensive monitoring by secondary psychiatry services; (2) primary diagnosis of bipolar disorder, obsessive-compulsive disorder, eating disorder, post-traumatic stress disorder or substance/alcohol misuse disorder; (3) taking warfarin; (4) having received tetracycline within the previous 2 months or having a history of sensitivity or intolerance to this class of drugs; (5) having an acute infection or an autoimmune or inflammatory disorder; (6) having hepatic or renal failure and (7) taking any other psychotropic medications other than their current antidepressant that has not been approved by a study investigator prior to enrolment. All female participants did a pregnancy test before starting the study and pregnant participants and those unwilling to use an acceptable form of contraceptive throughout the study period were also excluded.

Besides antidepressants (selective serotonin reuptake inhibitors, tricyclics, monoamine oxidase inhibitors, noradrenergic and specific serotonin antagonists and serotonin noradrenaline reuptake inhibitors), allowed medications included mood stabilizers and antipsychotics as long as patients were stable on treatment for at least 6 weeks at the time they entered the study. Participants undertaking psychotherapy and other psychosocial interventions were also included.

### Study procedure

After a screening visit to check their eligibility, participants (all outpatients) were invited to two further visits, the baseline and the week 4 visit (end of treatment). Within 1 month from the screening visit, eligible patients came back for the baseline visit and were randomized to minocycline or placebo (1:1) in addition to their current antidepressant. In the same day, we collected a blood sample for measurement of biological markers, including the inflammatory markers and the KP metabolites, and a clinical assessment including the Beck Depression Inventory II (BDI-II) (Kneipp et al., 2010). The same biological and clinical assessments were repeated at week 4 visit. Patients were provided with a diary to record their compliance to the daily dose of minocycline, any missed doses and side effects.

The final number of randomized patients was 44 (22:22). Five withdrew for different reasons (2 experienced side effects, 1 was lost in follow-up and 1 withdrew for unknown reasons in the minocycline group; 1 patient in the placebo group left for family issues); thus, the final sample consisted of 39 patients, 18 in the minocycline group and 21 in the placebo group. The consort flow diagram for RCTs is shown in our previous publication (Nettis et al., 2021).

### Immune biomarkers

At baseline and week 4, we analysed serum high-sensitivity (hs) CRP using a Roche Cobas 8000 (Mannheim, Germany) (von Eckardstein et al., 2013). Serum pro-inflammatory and anti-inflammatory cytokines, including IFN-γ, interleukin (IL)-1β, IL-2, IL-4, IL-6, IL-8, IL-10, IL-12p70, IL-13 and tumour necrosis factor alpha (TNF)-α were measured using Meso Scale Discovery (MSD) V-PLEX sandwich immunoassays, MSD Pro-inflammatory Panel 1 (human) kit (Dabitao et al., 2011; King et al., 2019) and plates read on an MSD QuickPlex SQ 120 (Rockville, Maryland, USA), as previously published (Hepgul et al., 2012; Russell et al., 2019). The inter-assay coefficient of variations was <10%. The results were analysed using MSD DISCOVERY WORKBENCH analysis software. Of note, levels of IL-1β, IL-4 and IL-12p70 were below the minimum detectable value for most of the subjects, so these cytokines were not included in the statistical analyses.

### KP metabolites

KP metabolites were analysed using plasma samples. The analytical method was adopted from Whiley et al. (2019) with modifications. Standard parent stock solution (1 mg/mL) and stable isotope-labelled stock solution (1 mg/mL) for targeted metabolites were prepared. Picolinic acid, picolinic acid-D_3_, QUIN, QUIN-D_3_, 3-HK, 3-HK-^13^C_2_-^15^N, kynurenine, kynurenine-D_4_, tryptophan and tryptophan-D_5_ were dissolved in 0.1% citric acid (1 mg/mL). KynA, KynA-D_5_, anthranilic acid, 3-hydroxyanthanilic acid and 3-hydroxyanthanilic acid-D_3_ were dissolved in 60% 0.1 M sodium hydroxide and 40% 1 mg/mL citric acid. The final concentrations of internal standard working solution are listed in Table 1.

**Table 1.** Final concentrations of internal working solution.

Internal standard	Concentration (ng/mL)
KynA-D_5_	60
Picolinic acid-D_3_	1500
QUIN-D_3_	1500
3-HK-^13^C_2_-^15^N	1500
Kynurenine-D_4_	1500
3-Hydroxyanthanilic acid-D_3_	1500
Tryptophan-D_5_	3000

3-HK: 3-hydroxykynurenine; KynA: kynurenic acid; QUIN: quinolinic acid.

For description of plasma extraction and preparation, see Supplemental Material.

The levels of KynA and anthranilic acid were below detectable threshold for, respectively, 81% and 50% of participants at baseline and 43% and 33% of participants at week 4, so we could not include them in the statistical analysis.

### Assessment of suicidal ideation

The presence (or absence) of suicidal ideation was measured using the item 9 of the BDI-II scale, a self-administered questionnaire. Answers to this item were coded 0 = *I don’t have any thoughts of killing myself*; 1 = *I have thoughts of killing myself, but I would not carry them out*; 2 = *I would like to kill myself*; 3 = *I would kill myself if I had a chance*. This item was used to create a dichotomic variable with 0 = *absence of suicidal ideation* (original score = 0) and 1 = *presence of suicidal ideation* (original score ⩾ 1). Of note, this single suicide item has been considered a valid approach to assess suicidal ideation by previous research (Desseilles, 2012). The BDI showed higher sensitivity than clinician-rated scales, like the HAM-D, in capturing suicidal ideation in prior work (Newport et al., 2007). This is particularly the case of outpatient clinical trials (Desseilles et al., 2012). Moreover, the BDI proved to have good concurrent and predictive validity in a number of studies, to be beneficial for measuring fluctuations in suicidal ideation throughout the course of treatment (Brown, 2001) and to have significant diagnostic accuracy for suicidal ideation and risk (Green et al., 2015).

### Statistical analysis

#### Baseline analysis

We performed Spearman correlations between peripheral inflammatory markers and kynurenine metabolites ratios indicating the progressive diversion of the KP towards the production of neurotoxic enzymes, in particular the KYN/TRP ratio, the 3HK/KYN ratio, the 3HAA/3HK ratio and the QUIN/3HK ratio. Correlations were corrected for multiple comparisons using Bonferroni. Then, with a Mann–Whitney test, we compared KP metabolites levels and ratios between patients with and without baseline suicidal ideation.

#### Longitudinal analysis

Repeated measures analysis of variance with KP as dependent variable and study arm (minocycline/placebo) and baseline suicidality (ideation present/absent) as factors. For this analysis, we log transformed KP variables to normalize their distribution. With a chi-square test, we explored differences in the proportion of patients with suicidal ideation between minocycline and placebo at baseline and week 4. Inflammatory cytokines were log transformed before conducting the analysis. Analyses were conducted on IBM SPSS Statistics version 26 (Armonk, NY, USA).

## Results

### Baseline data

#### KP activation in individuals with suicidal ideation

**Figure 1.**
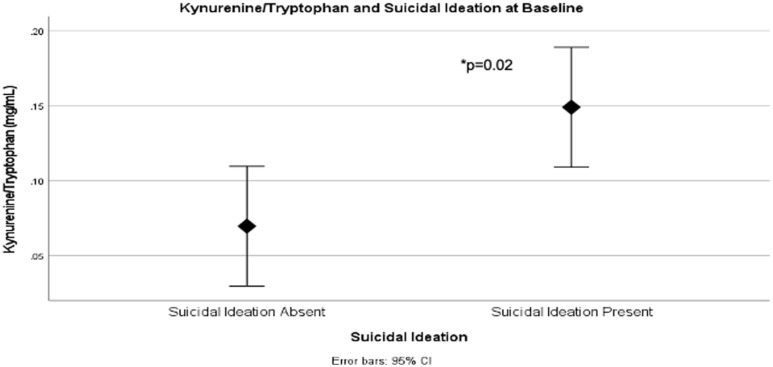
Mann–Whitney *U* test of KYN/TRP ratio in participants with (*N* = 22) and without (*N* = 22) baseline suicidal ideation. KYN/TRP ratio was significantly higher in suicidal participants. KYN: kynurenine; TRP: tryptophan.

We found significant differences in KYN/TRP ratio at baseline between patients with and without suicidal ideation as measured using the BDI-II. Indeed, those with baseline suicidal ideation showed significantly higher KYN/TRP ratio (*N* = 22, mean ± standard deviation (SD) = 0.15 ± 0.12) than those without suicidal ideation (*N* = 22, mean ± SD = 0.07 ± 0.05) (Mann–Whitney *U* = 143.000, *p* = 0.02) (see Figure 1).

#### Correlation between inflammatory markers and KP metabolites

In our sample at baseline (*N* = 44), higher levels of inflammatory markers were associated with higher KYN/TRP ratio and QUIN/3HK ratio. In particular, hsCRP levels and IL-10 levels were positively correlated with KYN/TRP ratio (Spearman’s ρ = 0.35, *p* = 0.02 and Spearman’s ρ = 0.41, *p* = 0.009, respectively). We also found a positive correlation of TNF with QUIN/3HK ratio (Spearman’s ρ = 0.55, *p* < 0.001). This last correlation survived Bonferroni correction for multiple comparisons.

### Longitudinal data

#### Effect of study arm on KP changes

When we explored the longitudinal effect of minocycline versus placebo in our sample of participants (number of participants who completed the study = 39), we found no significant difference between study arms in KP metabolites (and ratios) changes from baseline to week 4.

#### Effect of study arm on suicidal ideation

The chi-square analysis showed no difference between minocycline and placebo at baseline in terms of number of participants with/without suicidal ideation (Table 2). By contrast, at week 4, we found a difference at trend levels (χ^2^ = 2.7, *p* = 0.09), as illustrated in Table 3. In the minocycline group, the number of patients with suicidal ideation decreased by 50% (from being 44% of those on minocycline at baseline, to 22% at week 4).

**Table 2.** Number of participants in the minocycline and placebo groups with the absence or presence of suicidal ideation at baseline.

Baseline status of suicidal ideation	Number of participants (% within study arm)		Group difference		
	Minocycline	Placebo	χ^2^	df	*p* Value
	*N* *=* 18	*N* *=* 21			
Absence of suicidal ideation	10 (55.5%)	10 (47.6%)	0.24	1	0.43
Presence of suicidal ideation	8 (44.4%)	11 (52.4%)			

**Table 3.** Number of participants in the minocycline and placebo groups with the absence or presence of suicidal ideation at week 4.

Week 4 status of suicidal ideation	Number of participants (% within study arm)		Group difference		
	Minocycline	Placebo	χ^2^	df	*p* Value
	*N* *=* 18	*N* *=* 21			
Absence of suicidal ideation	14 (77.8%)	11 (52.4%)	2.7	1	0.09
Presence of suicidal ideation	4 (22.2%)	10 (47.6%)			

#### Effect of minocycline treatment and suicidal ideation on KYN pathway changes

**Figure 2.**
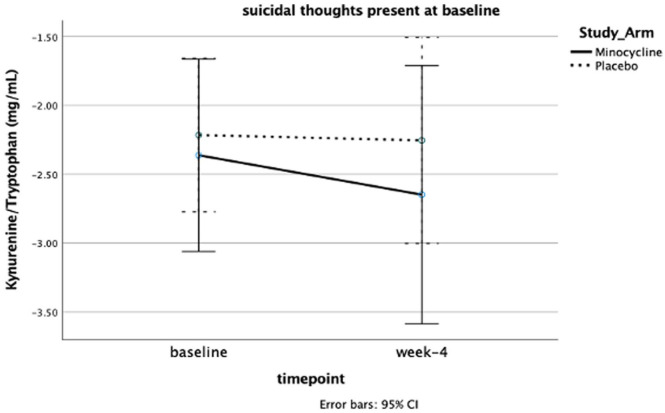
Repeated measures analysis of variance with log transformed KYN/TRP. In participants with suicidal ideation, the KYN/TRP slightly decreased in the minocycline group versus placebo, but without statistical significance. KYN: kynurenine; TRP: tryptophan.

Considering the increased baseline KYN/TRP for patients with suicidal ideation, we tested whether in these patients, study arm and baseline suicidal ideation influenced KP longitudinal changes. KYN/TRP appeared to overall decrease by week 4 in those with baseline suicidal ideation (from 0.14 ± 0.10 to 0.12 ± 0.08). However, we found no significant effect of study arm on such changes (Figure 2).

## Discussion

Our study showed that individuals with suicidal ideation have a higher KYN/TRP ratio than those without suicidal ideation and that higher levels of pro-inflammatory markers are associated with an activation of the neurotoxic branch of KP. Compared with placebo, in the minocycline group, the proportion of patients with suicidal ideation halved from baseline to week 4 (showing trend levels of significance, *p* = 0.09). However, such an outcome was not significantly associated with KP changes over time.

Our findings of an association between increased KP activation and suicidal ideation are consistent with previous studies conducted in MDD. Bradley et al. (2015) reported higher KYN/TRP ratio and lower levels of tryptophan in young MDD individuals with a history of a previous suicide attempt or with active suicidal ideation, when compared with non-suicidal subjects with depression and with healthy controls (Bradley et al., 2015). Additionally, Sublette et al. (2011) found that KYN levels were significantly higher amongst adults with MDD with a history of suicide attempt(s) in comparison with those with no suicide attempt(s) and healthy controls. Similarly, Messaoud et al. (2019) found that KYN/TRP ratio was more elevated, and levels of tryptophan were much lower in MDD patients with a history of suicide attempt(s) when compared with their non-suicidal counterparts. Results from the same study further revealed an association between increased KYN/TRP ratio and suicidal ideation measured by the Beck Scale for Suicidal Ideation (Beck et al., 1979). The present study, however, is the first to show an association between KP and suicidality in individuals specifically selected for elevated peripheral inflammation (CRP ⩾ 1 mg/L), which, in turn, might be contributing to the resistance to standard antidepressant treatment.

Several biological mechanisms have been suggested to link the KP to suicidal ideation. One hypothesis is that KP mediates the communication between peripheral and central inflammation, which has been suggested to play a role in the development of MDD suicidal ideation and has been observed in patients with suicidal history (Bryleva and Brundin, 2017b).

Moreover, the KP dysregulation induced by inflammatory processes leads to a depletion of serotonin and melatonin (Wislowska-Stanek et al., 2021) involved in mood and sleep–wake regulation, respectively. Finally, some KP metabolites, especially QUIN, might contribute to the pathophysiology of depression and suicidal behaviour. Erhardt et al. (2013) found that QUIN levels in the cerebrospinal fluid (CSF) of suicide attempters were statistically higher than QUIN levels in healthy controls (Erhardt et al., 2013). CSF QUIN positively correlated with suicidal intent and with CSF IL-6, confirming the role of inflammation in activating the neurotoxic branch of the KP. It has been hypothesized that the elevated levels of QUIN and its neurotoxic effects could contribute to the structural and functional changes observed in brain regions of psychiatric patients with suicidal behaviour. Indeed, Steiner and colleagues found an increase in microglial QUIN in the anterior cingulate cortex and anterior midcingulate cortex in post-mortem depressed patients who died of suicide (Steiner et al., 2011).

Consistent with some of the above-mentioned studies, we found an association between KP metabolites ratios indicating a neurotoxic diversion of the KP and inflammatory markers in our sample of patients at baseline. This is a further confirmation of the role of KP in linking inflammation to depression and suggests that a subpopulation of patients with immune-related depression who are non-responsive to standard antidepressants might benefit from KP-targeted investigations and treatment. This is also in line with previous clinical evidence of a correlation between increased peripheral inflammation and increased KP activation in depressed individuals. In particular, TNF and CRP have been found to be the inflammatory markers most closely associated with plasma concentrations of KYN and KYN/TRP (Haroon et al., 2020; Milaneschi et al., 2021). We also found a correlation between KYN/TRP and IL-10, which seems to have pleiotropic roles: it considered primarily an anti-inflammatory cytokine that could increase in response to KP activation to maintain homeostasis. However, recent evidence suggests it could also promote immune response (Bedke et al., 2019). Interestingly, previous studies found an association between IL-10 and suicidal ideation. For example, in a cross-sectional study of patients with MDD and controls, levels of IL-10 were higher in MDD patients with suicidal thoughts than non-suicidal MDD patients and controls (O’Donovan et al., 2013). Moreover, several studies found an increase in cytokines levels, including IL-6 and IL-10 in suicidal victims (Schizophrenia Working Group of the Psychiatric Genomics Consortium, 2014). This is in line with our findings that suicidal ideation is associated with higher KYN/TRP, which, in turn, is associated with higher IL-10 levels.

We noted that in patients with baseline suicidal ideation, KYN/TRP tended to decrease over time (although not significantly) in the minocycline group. Furthermore, by week 4, the minocycline group showed a reduction in the proportion of participants with suicidal ideation, compared with the placebo group (although this was also at trend levels). However, we could not find a significant effect of minocycline in affecting the KP metabolites, either in those with or in those without suicidal ideation. This could be due to the short duration of this trial (4 weeks). Indeed, our study did not detect robust changes in inflammatory markers from baseline to week 4 (Nettis et al., 2021), in contrast to previous longer trials of minocycline in depression (Savitz et al 2018). Thus, it is possible that after a longer treatment trial, the two longitudinal results of reduced suicidality and reduced KP dysregulation could fit together more clearly. It is also conceivable that longitudinal effects of minocycline on the KP were affected by the levels of baseline peripheral inflammation, in the same way that its antidepressant effect was limited to patients with CRP ⩾ 3 mg/dL in our previously published work.

Indeed, CRP levels ⩾ 3 mg/dL have been associated with no-response to standard antidepressants in patients with MDD (Miller and Raison, 2016) and with specific markers of central inflammation (Felger et al., 2020). However, the small sample size did not allow for a further stratification of patients based on both baseline suicidal ideation and CRP ⩾ 3 mg/dL. Finally, it has to be considered that the effect of minocycline in reducing suicidal ideation could also be mediated by other mechanisms besides the KP. For example, previous work found minocycline to react with a toxic and putatively psychotogenic catecholamine breakdown product, adrenochrome, removing it from solution. Interestingly, two KP intermediates, 3-OH-kynurenine and 3-OH-anthranilic acid, seem to inhibit the minocycline–adrenochrome reaction. Further work needs to clarify the effect of these two KP intermediate products on mental illness in the absence of minocycline and their role in modulating response to the same drug (Miller, 2015).

This study was limited by the small sample size. However, the secondary aims analyses were designed as explorative. Another limitation is that the levels of some KP metabolites (e.g., KynA) were below detectable threshold for most participants, so we could not include them in the analyses, even though we were able to identify important findings with the metabolites that were available. Finally, we excluded from the trial people with active and concerning suicidal ideation. This means that severely suicidal patients were not included, and that our subset of participants with suicidal ideation mainly had suicidal thoughts, without clear plans to act on them. This also means that our findings might reflect an association of increased KT ratio with the severity of depression, directly linked to suicidality, more than with suicidality per se. Unfortunately, we could not further explore this possibility in our sample, but future studies could attempt to clarify this. Indeed, it is possible that including participants with more severe suicidal ideation could lead to the identification of a stronger role of both KP and minocycline in suicidality. Finally, future studies might use more detailed questionnaires such as the Columbia Suicide Severity Rating Scale (CSSR-S) to capture different aspects of suicidality and its association with the KP.

## Conclusion

In conclusion, KP appears to play a role in increase suicidality in patients with depression and is associated with increased inflammation. Targeting KP in these individuals (especially those unresponsive to standard treatment) could be a potential personalized approach. Although minocycline appears to reduce suicidality in this population, further studies are needed to understand whether KP may be or not the mediating mechanism of this effect.

## Supplemental Material

sj-docx-1-jop-10.1177_02698811231173588 – Supplemental material for The interaction between kynurenine pathway, suicidal ideation and augmentation therapy with minocycline in patients with treatment-resistant depression Click here for additional data file. Supplemental material, sj-docx-1-jop-10.1177_02698811231173588 for The interaction between kynurenine pathway, suicidal ideation and augmentation therapy with minocycline in patients with treatment-resistant depression by Maria Antonietta Nettis, Giulia Lombardo, Caitlin Hastings, Zuzanna Zajkowska, Nicole Mariani, Naghmeh Nikkheslat, Luca Sforzini, Courtney Worrell, Amina Begum, Mollie Brown, Anthony J Cleare, Allan H Young, Carmine M Pariante and Valeria Mondelli in Journal of Psychopharmacology
