# Supplementary material for: The interaction between kynurenine pathway, suicidal ideation and augmentation therapy with minocycline in patients with treatment-resistant depression
Source: J Psychopharmacol. 2023 May 15;37(6):531–8. doi: 10.1177/02698811231173588 (PMC10291376; doi:10.1177/02698811231173588)
Supplement: sj-docx-1-jop-10.1177_02698811231173588 – Supplemental material for The interaction between kynurenine pathway, suicidal ideation and augmentation therapy with minocycline in patients with treatment-resistant depression [file sj-docx-1-jop-10.1177_02698811231173588.docx]

**Supplemental material**

## Kynurenine pathway analysis

## Plasma sample extraction and preparation

Plasma samples were left to thaw at 4 °C and then vortex mixed. An aliquot (50 μL) of each sample, calibration standard, quality control, and blank were transferred to 2mL Eppendorf tubes, and 20 μL of internal standard working solution was added to each Eppendorf tube except for double blank tubes.

Protein and phospholipid removal was performed using Phenomenex PHREE SPE cartridge (1mL, Phenomenex, Macclesfield, U.K.). Prior to use, the cartridges were prewashed using 500 μL of methanol containing 10 mM ammonium formate with a vacuum manifold. Samples were then loaded to the cartridges, washed with a further 500 μL of methanol containing 10 mM ammonium formate and eluted into collection Eppendorf tubes (2 mL).

The collection Eppendorf tubes were then taken to dryness with a Genevac EZ-2 -evaporation system for 2 hours (40 °C, “HPLC fraction” function). Dry extracts were resuspended in 40 μL of water containing 10 mM ammonium formate and 0.5% formic acid, and vortexed for 5 minutes before being transferred to HPLC vials. Five μL of each well was injected onto the UHPLC-ESI-MS/MS system for analysis.

## UHPLC-MS Analysis

The LC instrument setup consisted of a Waters Acquity UHPLC solvent management system and a Waters external autosampler (Waters, Wilmslow, U.K.). Chromatographic separation was performed with a Waters HSS T3 2.1 × 150 mm, 1.8 μm column (Waters, Wilmslow, U.K.). 0.1% formic acid in water (v/v) was used as mobile phase A, and 0.1% formic acid in acetonitrile (v/v) was employed as mobile phase B. Starting with 1%, mobile phase B content was increased to 10% over 3 min, then increased to 90% at 4 min, and finally returned to 1% at 4.1 min for column re-equilibration, which was completed at 5 min. The column temperature was maintained at 45 °C with a fixed flow rate of 0.6 mL/min.

MS detection was performed with a Waters Xevo Q-TOF quadrupole time of flight instrument (Waters, Wilmslow, U.K.) using electrospray ionization (ESI) in positive ionisation mode. Nitrogen was used as the desolvation gas, and argon was used as the collision gas. The following generic source conditions were applied: capillary voltage, 2.5 kV; sample cone, 30 V; extraction cone 2.0 V; source temperature, 150 °C; desolvation temperature, 600 °C; cone gas flow, 150 L/h; desolvation gas flow, 1000 L/h; collision gas, 7.0 bar.

## Data processing

Peak integration for raw spectral data was processed using Skyline software (V 21.1).
